# Fe^3+^ opposes the 1,25(OH)_2_D_3_-induced calcium transport across intestinal epithelium-like Caco-2 monolayer in the presence or absence of ascorbic acid

**DOI:** 10.1371/journal.pone.0273267

**Published:** 2022-08-30

**Authors:** Sukpapohn Phummisutthigoon, Kornkamon Lertsuwan, Nattapon Panupinthu, Ratchaneevan Aeimlapa, Jarinthorn Teerapornpuntakit, Wasutorn Chankamngoen, Jirawan Thongbunchoo, Narattaphol Charoenphandhu, Kannikar Wongdee

**Affiliations:** 1 Department of Physiology, Faculty of Science, Mahidol University, Bangkok, Thailand; 2 Center of Calcium and Bone Research (COCAB), Faculty of Science, Mahidol University, Bangkok, Thailand; 3 Department of Biochemistry, Faculty of Science, Mahidol University, Bangkok, Thailand; 4 Department of Physiology, Faculty of Medical Science, Naresuan University, Phitsanulok, Thailand; 5 Graduate Program in Molecular Medicine, Faculty of Science, Mahidol University, Bangkok, Thailand; 6 Institute of Molecular Biosciences, Mahidol University, Nakhon Pathom, Thailand; 7 The Academy of Science, The Royal Society of Thailand, Dusit, Bangkok, Thailand; 8 Faculty of Allied Health Sciences, Burapha University, Chonburi, Thailand; ICAR - National Dairy Research Institute, INDIA

## Abstract

Although iron is an essential element for hemoglobin and cytochrome synthesis, excessive intestinal iron absorption—as seen in dietary iron supplementation and hereditary disease called thalassemia—could interfere with transepithelial transport of calcium across the intestinal mucosa. The underlying cellular mechanism of iron-induced decrease in intestinal calcium absorption remains elusive, but it has been hypothesized that excess iron probably negates the actions of 1,25-dihydroxyvitamin D [1,25(OH)_2_D_3_]. Herein, we exposed the 1,25(OH)_2_D_3_-treated epithelium-like Caco-2 monolayer to FeCl_3_ to demonstrate the inhibitory effect of ferric ion on 1,25(OH)_2_D_3_-induced transepithelial calcium transport. We found that a 24-h exposure to FeCl_3_ on the apical side significantly decreased calcium transport, while increasing the transepithelial resistance (TER) in 1,25(OH)_2_D_3_-treated monolayer. The inhibitory action of FeCl_3_ was considered rapid since 60-min exposure was sufficient to block the 1,25(OH)_2_D_3_-induced decrease in TER and increase in calcium flux. Interestingly, FeCl_3_ did not affect the baseline calcium transport in the absence of 1,25(OH)_2_D_3_ treatment. Furthermore, although ascorbic acid is often administered to maximize calcium solubility and to enhance intestinal calcium absorption, it apparently had no effect on calcium transport across the FeCl_3_- and 1,25(OH)_2_D_3_-treated Caco-2 monolayer. In conclusion, apical exposure to ferric ion appeared to negate the 1,25(OH)_2_D_3_-stimulated calcium transport across the intestinal epithelium. The present finding has, therefore, provided important information for development of calcium and iron supplement products and treatment protocol for specific groups of individuals, such as thalassemia patients and pregnant women.

## Introduction

Daily iron and calcium requirement normally increases during pregnancy and lactation [1]. Since iron is believed to inhibit the intestinal calcium absorption, a combined calcium and iron supplementation is presently considered ineffective and not recommended [2–4]. In addition, there are certain conditions in which the intestinal iron absorption is markedly enhanced, for example, in a disease called thalassemia—a hereditary anemic disorder caused by globin gene mutation [5], in which calcium absorption may be compromised and bone disorder has been reported [6, 7]. Up until now, the underlying cellular mechanism of iron-induced inhibition of calcium absorption has been elusive. Since cellular uptake of iron and calcium occurs through completely different sets of transporting proteins (please see below), it is unlikely that iron interfere directly with transepithelial transport of calcium. Therefore, we hypothesized that iron probably hinders the stimulatory effect of 1,25-dihydroxyvitamin D_3_ [1,25(OH)_2_D_3_] on calcium absorption.

Under normal conditions, 1,25(OH)_2_D_3_ enhances cellular calcium uptake across the apical membrane of enterocytes by upregulating the expression and activity of divalent ion channel transient receptor potential vanilloid subfamily member 6 (TRPV6) [8]. Meanwhile, it also accelerates the cytoplasmic calcium translocation and plasma membrane Ca^2+^-ATPase-1b (PMCA_1b_)-mediated calcium extrusion across the basolateral membrane [8]. In other words, 1,25(OH)_2_D_3_ exerts its positive effects on all steps of the transcellular calcium absorption, particularly in the proximal small intestine (duodenum and proximal jejunum) and proximal large intestine (cecum) [8–10]. Although there are several factors that potentially reduce intestinal calcium absorption, such as calcitonin and stanniocalcin [11–13], only a few have been reported to diminish the 1,25(OH)_2_D_3_-induced calcium absorption. For example, fibroblast growth factor (FGF)-23—either from the systemic circulation or local cellular production—is a known inhibitory factor for 1,25(OH)_2_D_3_ signaling as well as the 1,25(OH)_2_D_3_-stimulated calcium absorption [14, 15]. Besides stimulating the transcellular calcium transport, 1,25(OH)_2_D_3_ also enhances calcium movement across the paracellular pathway by reducing the intercellular resistance and increasing tight junction permselectivity, which represents an ability of the intestinal epithelium to discriminate ions with different size and charge, including calcium [8]. Thus, in the presence of high-calcium concentration in the intestinal lumen, 1,25(OH)_2_D_3_ is able to upregulate both transcellular and paracellular calcium transports and becomes an important regulator of calcium absorption.

Dietary compositions, such as oxalate, phytate, quercetin, and iron can modulate intestinal absorption of minerals [16, 17]. It is well established that iron is normally transported across the apical and basolateral membrane of enterocyte by divalent metal transporter (DMT)-1 and ferroportin-1, respectively [18], and iron transport mechanism is probably not directly related to that of calcium uptake. Hence, the explanations of iron-induced inhibition of calcium transport are often based on iron/calcium physicochemical interaction in aqueous environment, change in calcium solubility, or an increase in cellular free radical production, the last of which was reported to reduce intestinal calcium transport [19–21]. On the other hand, other molecules, e.g., ascorbic acid, has long been used to increase calcium solubility and reduce cellular oxidative stress, but whether it can promote calcium absorption in the presence of iron remains unclear. Nevertheless, the fact that 1,25(OH)_2_D_3_ is the salient stimulator of calcium absorption ushers us to postulate that iron, by compromising 1,25(OH)_2_D_3_ action, is probably a potent inhibitor of the 1,25(OH)_2_D_3_-induced calcium transport.

Therefore, the objectives of the present study were (*i*) to investigate the effects of ferric ion (Fe^3+^) from iron(III) chloride (FeCl_3_) on the transepithelial calcium transport across the intestinal epithelium-like Caco-2 monolayer with or without 1,25(OH)_2_D_3_ pre-treatment, (*ii*) to determine the acute response of Caco-2 cells to ferric ion exposure, and (*iii*) to demonstrate whether ascorbic acid was able to revert the action of ferric ion. Under normal conditions, DMT1 transports only Fe^2+^; therefore, Fe^3+^ used in the present experiment (i.e., FeCl_3_) must be reduced to Fe^2+^ by Dcytb (ferric reductase) prior to apical uptake by DMT1. In addition, FeCl_3_ has previously been used to study iron uptake in Caco-2 cells [22]. Furthermore, we avoided using iron salts consisting of anions with ≥2 negative charges (e.g., sulfate, citrate or ethylenediaminetetraacetate) since the anions may bind to or form insoluble complexes with Ca^2+^. Caco-2 monolayer was used in the present study because it has been shown to have functional characteristic of small intestine, including expression of transcellular calcium transporters (e.g., TRPV6 and calbindin-D_9k_), presence of the brush border, expression of sucrase-isomaltase enzyme, and responses to vitamin D [23–25].

## Materials and methods

### Cell culture

Intestinal epithelium-like Caco-2 cells obtained from American Type Culture Collection (ATCC no. HTB-37; RRID CVCL_0025) were grown in Dulbecco’s modified Eagle’s medium (DMEM) (Sigma, St. Louis, MO, USA) supplemented with 15% fetal bovine serum (FBS) GIBCO, Grand Island, NY), 1% L-glutamine (GIBCO), 1% non-essential amino acid (Sigma), 100 U/mL penicillin-streptomycin (Sigma), and 0.25 μL/mL amphotericin B (Sigma). Cells were propagated in a 75-cm^2^ T flask (Corning, NY, USA) under humidified atmosphere containing 5% CO_2_ at 37°C and subcultured as described in the ATCC’s protocol. Thereafter, Caco-2 cells (420,000 cells/well) were grown on a porous polyester membrane, i.e., Snapwell with a diameter of 12 mm and pore size of 0.4 μm (catalog no. 3801; Corning), as reported previously [26]. Culture media was changed daily, and monolayers were incubated at 37°C for 3 days in a humidified atmosphere containing 5% CO_2_. Under normal conditions, Caco-2 cells that form a confluent monolayer will develop microvilli and tight junction with abundant expression of calcium-transporting proteins, e.g., TRPV6, calbindin-D_9k_ and PMCA_1b_, similar to the small intestinal epithelial cells [24, 27].

### Experimental design

Unless otherwise specified, Caco-2 monolayers were incubated with culture media containing 0, 1, 10 or 100 nM 1,25(OH)_2_D_3_ (catalog no. 71820; Cayman Chemical, MI, USA) on both apical and basolateral compartments for 72 h. Thereafter, each Snapwell was transferred into Ussing chamber for determination of transepithelial calcium flux and epithelial electrical parameters. To demonstrate the negative effect of ferric ion on 1,25(OH)_2_D_3_-induced transepithelial calcium transport, the 1,25(OH)_2_D_3_-treated monolayers were exposed for 24 h to 100 μM FeCl_3_ in the basolateral compartment (catalog no. 157740; Sigma-Aldrich, Saint Louis, MO, USA).

In some experiments, Caco-2 monolayers were pre-incubated for 24 h with 0.5 mM ascorbic acid (catalog no. A8960; Sigma-Aldrich, Saint Louis, MO, USA) to demonstrate whether ascorbic acid was able to counterbalance the action of ferric ion on calcium transport. The concentration ranges of FeCl_3_ and ascorbic acid were the optimal concentration without causing toxicity to the cells and consistent with previous reports [22, 28]. FeCl_3_ treatment protocol was sub-divided into (*i*) an acute exposure protocol, in which FeCl_3_ was directly added into the apical hemichamber during Ussing chamber study, and (*ii*) a prolonged exposure protocol, in which Caco-2 monolayers were incubated in culture media containing FeCl_3_ in both apical and basolateral compartments. We added FeCl_3_ in the basolateral compartment to ensure that even though a prolonged exposure to Fe^3+^ might increase extracellular Fe^3+^ concentration in the close vicinity to the basolateral membrane, it could not decrease the baseline calcium flux. In other words, in a condition with high serum free iron, it was likely affected the 1,25(OH)_2_D_3_-induced calcium flux rather than the baseline calcium flux.

### Measurement of transepithelial calcium transport using radioactive tracer

Ussing chamber technique was used to determine the transepithelial calcium flux, as previously described [7]. In brief, Caco-2 monolayer was first mounted and equilibrated between apical and basolateral hemichambers for 10 min in isotonic bathing solution, which was comprised of (in mM) 118 NaCl, 4.7 KCl, 1.1 MgSO_4_, 1.25 CaCl_2_, 23 NaHCO_3_, 12 D-glucose and 2 mannitol (all purchased from Sigma). The solution was continuously gassed all the time with humidified 5% CO_2_ in 95% O_2_, and maintained at 37°C and pH 7.4. The osmolality was 290–293 mmol/kg water as measured by a freezing point-based osmometer (model 3320; Advanced Instruments, Norwood, MA, USA). Thereafter, the bathing solution in the apical hemichamber was replaced with fresh bathing solution containing ^45^Ca at the initial amount of 0.451 Ci/mL and final specific activity of 90 mCi/mol (catalog no. NEZ013; PerkinElmer, Boston, MA, USA), while the basolateral side was replaced with fresh normal bathing solution. The ^45^Ca radioactivity in counts per minute was analyzed by a liquid scintillation spectrophotometer (model Tri-Carb 3100; Packard, Meriden, CT, USA). Radiotracer samples were collected from Ussing chamber, and the unidirectional calcium flux in the apical-to-basolateral direction was calculated as previously described [26].

### Measurement of epithelial electrical parameters

The epithelial electrical parameters, i.e., transepithelial potential difference (PD or voltage), short-circuit current (*I*_sc_) and transepithelial resistance (TER), were determined as described previously [6]. In brief, PD and *I*_sc_ were recorded by two pairs of electrodes made of Ag/AgCl half cells connecting with Ussing chamber through salt bridges (2 M KCl in 3 g% agar). The PD-sensing electrodes were placed near the Caco-2 monolayer, connected to a preamplifier (model EVC-4000; World Precision Instruments, Sarasota, FL, USA) and PowerLab digital recording system (model 4/30; ADInstruments, Colorado Springs, CO, USA). An *I*_sc_-passing electrode was located at the rear end of each hemichamber, connected in series to the EVC-4000 current-generating unit and PowerLab 4/30 operated with Chart version 5.2.2. Fluid resistance was subtracted by the EVC-4000 system. TER was calculated from Ohm’s equation.

### Quantitative real-time PCR

The mRNA expression levels of ascorbic acid transporters (SVCT1 and SVCT2), TRPV6, calbindin-D_9k_, PMCA_1b_ and DMT1 in Caco-2 monolayers were measured by real-time PCR. Total RNA was prepared by using TRIzol extract reagent (Invitrogen, Carlsbad, USA), as previously described [29]. Total RNA concentration was determined by NanoDrop-2000c spectrophotometer (Themo Specific, Waltham, MA, USA) and the 260/280-nm ratio ranged 1.8–2.0. One microgram of total RNA was then reverse-transcribed into cDNA by iScript cDNA synthesis kit (Bio-rad, Hercules, CA, USA). PCR and melting curve analyses were operated by QuantStudio 3 real-time PCR system (Applied Biosystems, MA, USA) with glyceraldehyde-3-phosphate dehydrogenase (housekeeping gene) or other primers (Table 1). The mRNA expression levels were calculated based on the method of Livak and Schmittgen [30].

**Table 1:** *Homo sapiens* primers used in real-time PCR.

Gene	Accession no.	Primer (Forward/Reverse)	Product size (bp)	Annealing temperature (°C)
*Vitamin C transporters*				
SVCT1	NM_005847	5’–TCATCCTCTTCTCCCAGTACCT–3’	141	57
		5’–AGAGCAGCCACACGGTCAT–3’		
SVCT2	NM_005116	5’–GCACCCAGCTTTCTTCACTC–3’	163	61
		5’–CAGACTGCCAGTGCTATCCA–3’		
*Calcium transport related genes*				
TRPV6	AF365928	5’–TCTGACTGCGTGTTCTCAC–3’	144	56
		5’–ACATTCCTTGGCGTTCAT–3’		
Calbindin-D_9k_	NM_004057	5’–TAGCTGTTTCACTATTGGGCA–3’	127	56
		5’–TTCATCCTTTGACAACTGGTCT–3’		
PMCA_1b_	NM_001001323	5’–AGAAGGTGGAGATGGTGATGA–3’	179	56
		5’–CCCAGAAGGTGTCAATGACA–3’		
*Iron transporter*				
DMT1	NM_001174125	5’–CTTTGCCCGAGTGGTTCTGA–3’	185	56
		5’–AGTCACTCATTACTGGCCGC–3’		
*Housekeeping gene*				
GAPDH	NM_001289746	5’–TTGTTGCCATCAATGACCC–3’	166	53
		5’–ATTTTGGAGGGATCTCGCT–3’		

SVCT1, solute carrier family 23 member 1 (SLC23A1); SVCT2, solute carrier family 23 member 2 (SLC23A2); TRPV6, transient receptor potential cation channel subfamily V member 6; PMCA_1b_, plasma membrane Ca^2+^-ATPase-1b; DMT1, divalent metal transporter-1; GAPDH, glyceraldehyde-3-phosphate dehydrogenase.

### Cell viability assay

Viability of Caco-2 cells treated with various concentrations of FeCl_3_ was assessed by using 3-(4,5-dimethylthiazol-2-yl)-2,5-diphenyltetrazolium bromide (MTT) colorimetric assay. In brief, Caco-2 cells were plated in 96-well plate at 25,000 cells/well for 24 h, and were then treated with FeCl_3_ at concentrations ranging from 0 to 200 μM for 24, 48 and 72 h. To assess cell viability, the MTT solution (catalog no. M5655; Sigma) was added to obtain a final concentration of 0.5 mg/mL for 4 h to generate formazan crystals, which were dissolved with dimethyl sulfoxide. The color was measured at the absorbance of 540 nm with a microplate spectrophotometer.

### Statistical analysis

The results are expressed as means ± standard errors. Two-group data were compared by unpaired Student’s *t*-test. One-way analysis of variance (ANOVA) with Tukey’s multiple comparison test was used for multiple sets of data. All analyses were performed by using GraphPad Prism 9 (GraphPad Software Inc., San Diego, CA, USA). The level of significance for all statistical tests was *P* < 0.05.

## Results

**Fig 1:**
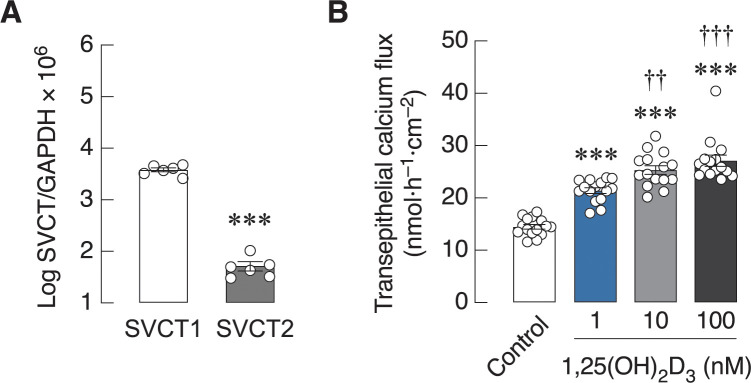
mRNA expression of vitamin C transporters and 1,25(OH)_2_D_3_-induced calcium transport in Caco-2 monolayer. Expression of sodium-vitamin C co-transporters (SVCT and SVCT2) in Caco-2 cells (A). GAPDH is a housekeeping gene for normalization. (n = 6; ***, *P* < 0.001 compared with the SVCT1 group. (B) Transepithelial calcium flux across the 1,25(OH)_2_D_3_-treated Caco-2 monolayers in Ussing chamber in the absence of transepithelial calcium gradient. ***P < 0.001 compared with the control group (white bar); ^††^*P* < 0.01; ^†††^*P* < 0.001 compared with the 1 nM 1,25(OH)_2_D_3_-treated group (blue bar).

Prior to investigating the effects of FeCl_3_ on the 1,25(OH)_2_D_3_-induced calcium transport, the Caco-2 cells were verified for the normal response to 1,25(OH)_2_D_3_ and the expression of sodium-vitamin C co-transporters (i.e., SVCT1 and SVCT2), which are essential for cellular ascorbic acid uptake [31, 32]. Quantitative real-time PCR analysis showed that Caco-2 cells were able to express both SVCT1 and SVCT2 transcripts with the mRNA level of SVCT1 being greater than that of SVCT2 (Fig 1A). Moreover, after exposure to 1, 10 or 100 nM 1,25(OH)_2_D_3_, the transepithelial calcium fluxes were significantly enhanced across the Caco-2 monolayers in a dose-dependent manner (Fig 1B). Since we performed the Ussing chamber experiment in an absence of transepithelial calcium gradient—i.e., both apical and basolateral hemichambers contained equal free-ionized calcium concentration of 1.25 mM, the observed calcium flux represented the transcellular calcium transport in an apical-to-basolateral direction. We also verified that Caco-2 cells responded to 1,25(OH)_2_D_3_ by increasing the transcellular calcium transport, similar to that observed in the proximal small intestine [9].

**Fig 2:**
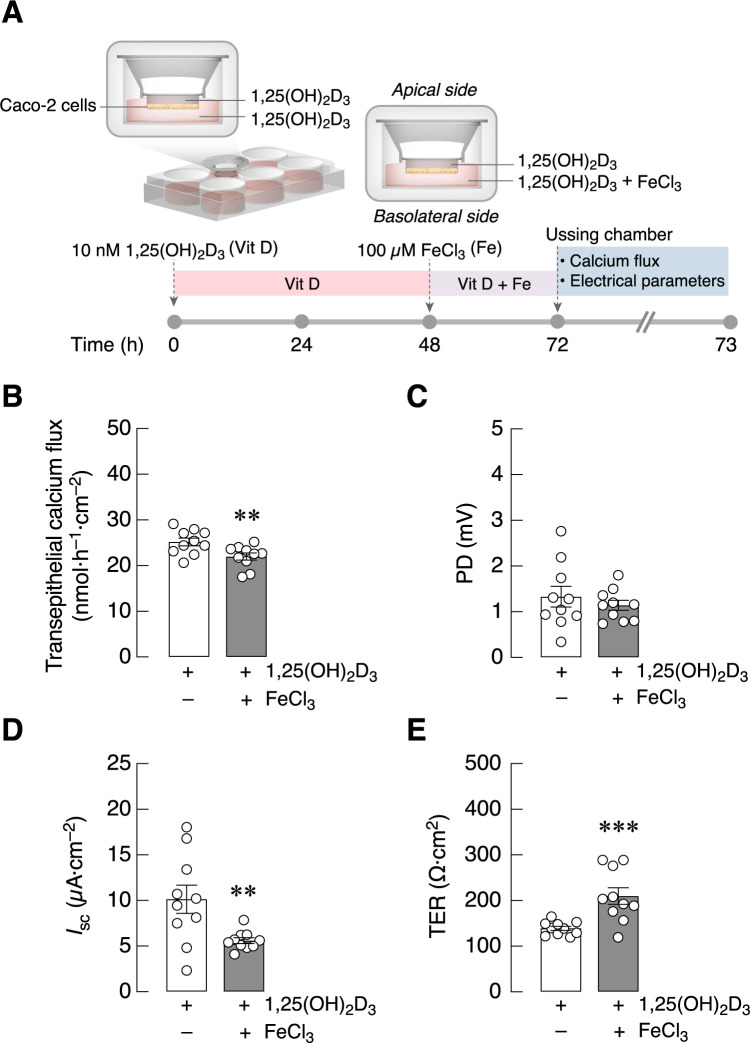
Transepithelial calcium flux of FeCl_3_ and 1,25(OH)_2_D_3_-treated Caco-2 monolayer. (A) Experimental timeline of 1,25(OH)_2_D_3_ and FeCl_3_ treatment (please see text for detail). (B–E) Transepithelial calcium transport and epithelial electrical parameters (PD, *I*_sc_, and TER) in 1,25(OH)_2_D_3_-treated Caco-2 monolayers with or without 100 μM FeCl_3_. PD values were the magnitudes of potential difference (the apical side being negative with respect to the basolateral side), and glucose made the apical side more negative. (n = 10; **P < 0.01; ***P < 0.001 compared with the control group (white bar).

Thereafter, a series of experiments was performed to demonstrate that 24-h exposure to ferric ion had an inhibitory effect on calcium transport across Caco-2 monolayer pre-treated with 10 nM 1,25(OH)_2_D_3_ for 72 h (Fig 2A). The results revealed that, despite the absence of both 1,25(OH)_2_D_3_ and FeCl_3_ in Ussing chamber, the transepithelial calcium flux of FeCl_3_ and 1,25(OH)_2_D_3_-treated monolayer was less than that of the monolayer treated with 1,25(OH)_2_D_3_ alone (Fig 2B). Meanwhile, FeCl_3_ significantly decreased *I*_sc_ and increased TER with no effect on PD (Fig 2C–2E). Thus, the actions of 1,25(OH)_2_D_3_ and FeCl_3_ during the pre-treatment of the Caco-2 cells persisted although Caco-2 cells in Ussing chamber no longer exposed to both agents.

**Fig 3:**
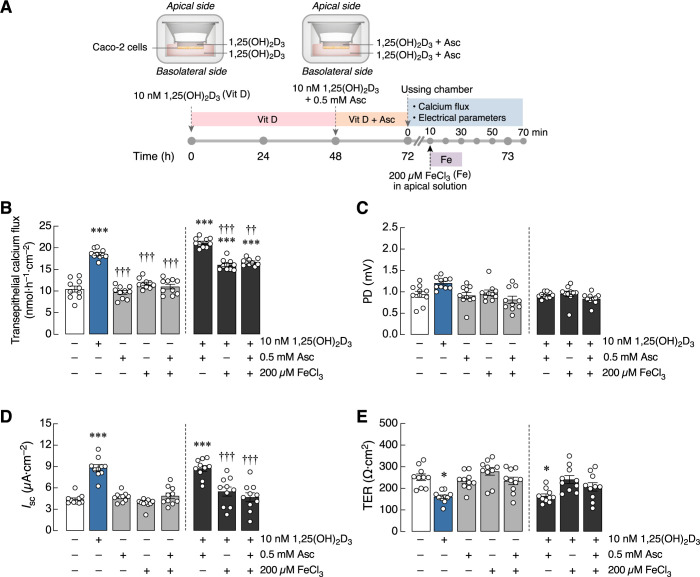
The role of FeCl_3_ on calcium transport across Caco-2 monolayer pre-treated with 1,25(OH)_2_D_3_ and ascorbic acid (Asc). (A) Experimental timeline (please see text for detail). (B–E) Transepithelial calcium transport and epithelial electrical parameters (PD, *I*_sc_, and TER) in Caco-2 monolayers with or without 10 nM 1,25(OH)_2_D_3_, 200 μM FeCl_3_, and 0.5 mM Asc. PD values were the magnitudes of potential difference (the apical side being negative with respect to the basolateral side), and glucose made the apical side more negative. (n = 10; *P < 0.05; ***P < 0.001 compared with the control group (white bar); ^††^*P* < 0.01, ^†††^*P* < 0.001 compared with the 10 nM 1,25(OH)_2_D_3_-treated group (blue bar).

In Fig 3A, we further explored whether acute exposure to ferric ion in Ussing chamber was capable of diminishing calcium transport in Caco-2 monolayer pre-treated for 72 h with 1,25(OH)_2_D_3_, and whether ascorbic acid pre-treatment could revert the diminished calcium flux. As depicted in Fig 3B, FeCl_3_ significantly decreased calcium transport in 10 nM 1,25(OH)_2_D_3_-treated Caco-2 monolayer, but not in monolayer without 1,25(OH)_2_D_3_ treatment. FeCl_3_ also reverted the 1,25(OH)_2_D_3_-induced changes in *I*_sc_ and TER to control levels [i.e., cells without 1,25(OH)_2_D_3_ and FeCl_3_], with no PD changes (Fig 3C–3E). However, ferric ion did not affect the epithelial electrical parameters of cells without 1,25(OH)_2_D_3_ treatment (Fig 3C–3E). In addition, although ascorbic acid has been known to increase the solubility of calcium compounds, as shown in the present results (S1 Fig), 24 h ascorbic acid pre-treatment did not alter the electrical parameters or transepithelial calcium transport across Caco-2 monolayer with or without exposure 1,25(OH)_2_D_3_ (Fig 3).

**Fig 4:**
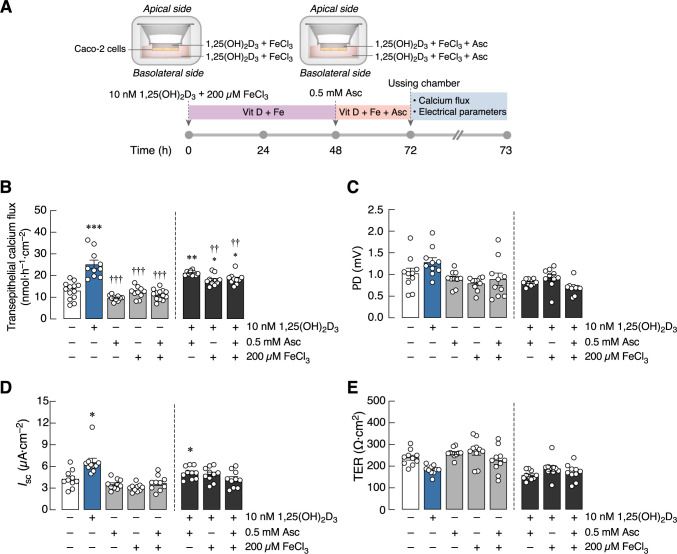
Calcium transport across Caco-2 monolayer combine treated with 1,25(OH)_2_D_3_, ascorbic acid (Asc), and FeCl_3_. (A) Experimental timeline (please see text for detail). (B–E) Transepithelial calcium transport and epithelial electrical parameters (PD, *I*_sc_, and TER) in Caco-2 monolayers with or without 10 nM 1,25(OH)_2_D_3_, 200 μM FeCl_3_, and 0.5 mM Asc. PD values were the magnitudes of potential difference (the apical side being negative with respect to the basolateral side), and glucose made the apical side more negative. (n = 10; *P < 0.05; **P < 0.01; ***P < 0.001 compared with the control group (white bar); ^††^*P* < 0.01; ^†††^*P* < 0.001 compared with the 10 nM 1,25(OH)_2_D_3_-treated group (blue bar).

We further investigated whether the inhibitory action of FeCl_3_ would still be observed after a prolonged 72 h exposure to ferric ion, or whether cells could eventually adapt to prolonged high-iron milieu by decreasing the inhibitory action of FeCl_3_ and maintaining the 1,25(OH)_2_D_3_-induced calcium transport (Fig 4A). As shown in Fig 4B, 72-h FeCl_3_ exposure was able to diminish the 1,25(OH)_2_D_3_-induced calcium transport. Nevertheless, the FeCl_3_ action on *I*_sc_ and TER was trivial compared to that observed in the acute FeCl_3_ exposure experiment (Fig 4C–4E). Similar to the earliest experiment, ascorbic acid showed no effect on either epithelial electrical parameters or calcium transport (Fig 4B–4E).

**Fig 5:**
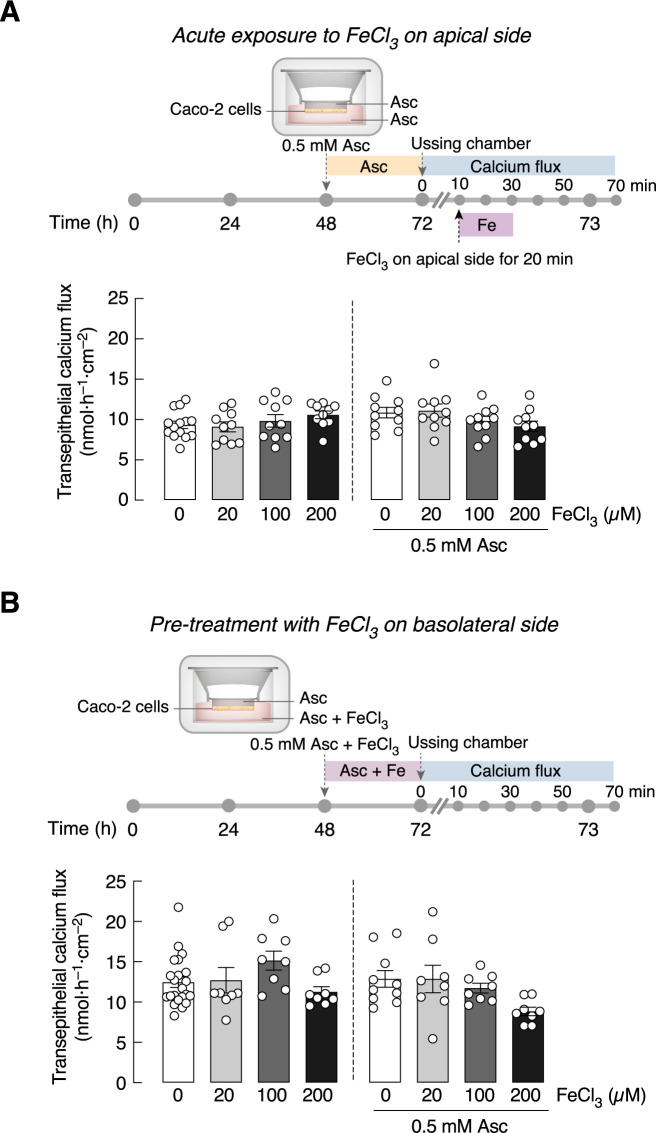
Acute and long-term effects of FeCl_3_ exposure on calcium transport in Caco-2 monolayer pre-treated with ascorbic acid (Asc). (A) Transepithelial calcium transport across Caco-2 monolayers with or without 0.5 mM Asc pre-treatment and acute exposure with different doses of FeCl_3_ (i.e., 0, 20, 100, 200 μM) on apical side. (B) Transepithelial calcium transport across Caco-2 monolayers with or without 0.5 mM Asc with different doses of FeCl_3_ (i.e., 0, 20, 100, 200 μM) pre-treatment on basolateral sides (n = 10).

**Fig 6:**
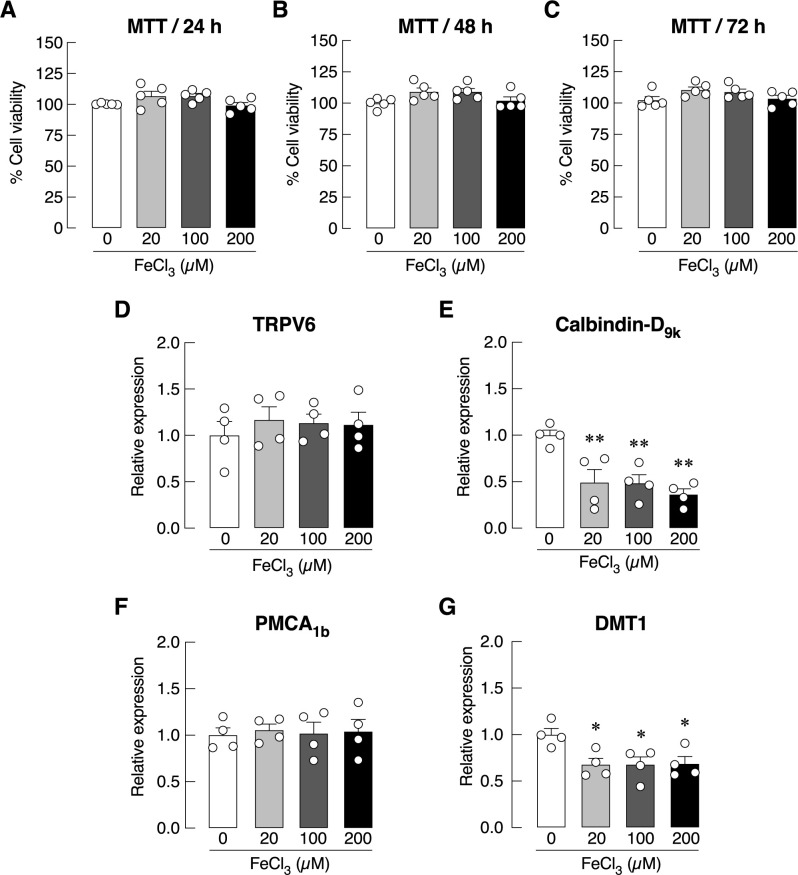
Viability and mRNA expression of calcium transport-related genes and DMT1 gene in Caco-2 cells treated with FeCl_3_. (A–C) Cell viability of Caco-2 cells treated with various concentrations of FeCl_3_ (i.e., 0, 20, 100, 200 μM) for 24, 24 and 72 h. (D–G) mRNA expression of calcium transport-related genes (i.e., TRPV6, calbindin-D_9k_, PMCA_1b_), and iron transporter DMT1 gene in Caco-2 cells treated with various concentrations of FeCl_3_ (i.e., 0, 20, 100, 200 μM) for 24 h.

The last series of experiments aimed to demonstrate whether FeCl_3_ affect baseline calcium flux in the presence or absence of ascorbic acid, Caco-2 monolayers were pre-incubated for 24 h with 0.5 mM ascorbic acid in both apical and basolateral compartments. The results confirmed that acute exposure to 20, 100 or 200 μM FeCl_3_ in Ussing chamber—either on the apical or basolateral side—did not affect the baseline calcium transport (Fig 5A and 5B). In addition, ascorbic acid pre-treatment did not alter the transepithelial calcium transport across the Caco-2 monolayer. As shown in Fig 6, exposure to 20, 100 or 200 μM FeCl_3_ for 24–72 h did not affect Caco-2 cells viability or the mRNA levels of TRPV6 and PMCA_1b_. Nevertheless, FeCl_3_-exposed Caco-2 cells exhibited downregulation of calbindin-D_9k_ and DMT1 mRNA expression.

## Discussion

Under normal conditions, calcium and bone metabolism is tightly regulated by several hormones, e.g., 1,25(OH)_2_D_3_, estrogen and prolactin [29, 33–35]. Regarding intestinal calcium uptake, ~15–30% of dietary calcium is absorbed into the circulation [34, 36]. In other words, after ingesting 1,000 mg/day elemental calcium, the net intestinal calcium uptake into the body is ~150–300 mg/day. This relatively low fractional calcium absorption is often thought to be due to low transporting capacities of the apical calcium channels and/or basolateral calcium transporters rather than the presence of calcium transport inhibitors—such as iron or FGF-23 [2, 3, 14, 15]. Herein, we elaborated the inhibitory effect of ferric ion on the 1,25(OH)_2_D_3_-induced transcellular calcium transport across the intestinal epithelium-like Caco-2 monolayer. Since the present data showed that ferric ion reverted both transcellular calcium flux and electrical parameters (i.e., *I*_sc_ and TER), which indicated paracellular permeability, it was unlikely that ferric ion directly inhibited the transcellular calcium transporters, but it probably compromised overall 1,25(OH)_2_D_3_ actions in a similar manner to those of other inhibitory factors, such as FGF-23 (for review, please see [37]).

As mentioned earlier, 1,25(OH)_2_D_3_ has been known to stimulate every step of the intestinal transcellular calcium transport—i.e., TRPV6-mediated apical calcium influx, calbindin-D_9k_-assisted cytoplasmic calcium translocation and PMCA_1b_-mediated basolateral calcium efflux [8–10]. Therefore, blockade of 1,25(OH)_2_D_3_ action almost abolishes the transcellular calcium transport [38, 39]. We have previously demonstrated that FGF-23 was capable of downregulating the 1,25(OH)_2_D_3_-induced transcellular calcium transport across mouse intestinal epithelium and Caco-2 monolayer as well as the expression of calbindin-D_9k_, which was considered a cellular biomarker of 1,25(OH)_2_D_3_ repletion [14, 15, 26]. Because FGF-23 activates the intracellular catabolism of 1,25(OH)_2_D_3_ by upregulating the 24-hydroxylase expression [37, 40], the presence of FGF-23 would reduce the cytoplasmic level of 1,25(OH)_2_D_3_, thereby reducing its binding to vitamin D receptor (VDR). It is noteworthy that enterocytes, including Caco-2 cells, do express FGF-23, which probably helps prevent excessive calcium absorption during 1,25(OH)_2_D_3_ stimulation [26, 41].

As depicted in Fig 6, ferric ion did not directly affect the mRNA levels of TRPV6 and PMCA_1b_. However, downregulation of calbindin-D_9k_ mRNA expression might somewhat deteriorate capability of Caco-2 cells to translocate intracellular calcium ions, but this genomic or transcriptional change was not large enough to alter calcium flux (Fig 5), consistent with the existence of transcellular calcium transport in calbindin-D_9k_ knockout mice [42]. Although the exact cellular and molecular mechanism(s) of ferric ion-induced inhibition of the vitamin D-stimulated calcium transport remains elusive, cellular oxidative stress induced by cellular iron uptake and the resultant reactive oxygen species (ROS) production could be at least partially responsible for the inhibitory effect of the ferric ion on 1,25(OH)_2_D_3_-induced calcium transport. More evidence supporting the impact of oxidant-antioxidant balance on cellular function was provided by experiment in rat renal proximal tubular cells. Hydrogen peroxide, which is ROS, was found to upregulate 24-hydroxylase expression [43], leading to an increase in intracellular 1,25(OH)_2_D_3_ degradation. ROS not only impaired VDR, but also suppressed transcriptional activation of retinoic acid receptor/retinoid X receptor (RXR) [44], which forms a heterodimer and translocates to interact with specific vitamin D response elements (VDREs) in vitamin D-responsive genes [45]. Acute and prolonged exposure to pro-oxidants, such as menadione, is also known to directly inhibit mitochondrial function and cellular energy-dependent calcium transporters [20]. Furthermore, we previously provided evidence that, in thalassemic mice with intestinal iron hyperabsorption, iron could interfere with the cytoplasmic vesicular calcium uptake, thereby slowing down the transcellular calcium transport across the small intestinal epithelium [7].

Interestingly, the epithelial electrical parameters, *I*_sc_ and TER, were also altered by 1,25(OH)_2_D_3_. The absence of PD changes suggested that an increase in *I*_sc_ might have resulted from an increased paracellular permeability rather than the increased electrogenic ion transport. TER apparently decreased under 1,25(OH)_2_D_3_-exposed conditions, consistent with an increase in *I*_sc_. A decrease in TER indeed favors paracellular calcium movement. Specifically, in the presence of transepithelial calcium gradient (e.g., high luminal calcium concentration during calcium supplementation), 1,25(OH)_2_D_3_ is able to enhance the paracellular calcium absorption by increasing expression of claudin-2 and -12. Both claudins normally form cation-selective tight junction pores, thereby enhancing paracellular cation movement as represented by a reduction in TER, and increasing tight junction permeability to calcium as well [46]. It was herein apparent that ferric ion negated 1,25(OH)_2_D_3_ action, thus reverting *I*_sc_ and TER to the control levels. In other words, it was likely that ferric ion exposure was able to reduce the paracellular transport of calcium and some other cations (e.g., sodium), as indicated by greater TER and lower *I*_sc_ in 200 μM FeCl_3_+10 nm 1,25(OH)_2_D_3_ group vs. 10 nm 1,25(OH)_2_D_3_ alone (Fig 3D and 3E). Although cellular oxidative stress could exert a negative effect on tight junction and paracellular calcium transport [20], cellular oxidative stress due to iron exposure in this study did not alter the epithelial electrical parameters in the absence of 1,25(OH)_2_D_3_. Therefore, ferric ion and/or ROS predominantly interfered with 1,25(OH)_2_D_3_ action rather than producing a direct effect on the tight junction function.

Besides having many health benefits such as being an anti-oxidant, ascorbic acid is able to increase the solubility of certain calcium compounds, such as calcium carbonate (S1 Fig); therefore, it was often added in calcium supplement formulations to help accrue free-ionized calcium in the intestinal lumen. In human and rodent intestine, luminal calcium must be solubilized into free-ionized form before being absorbed into the body via transcellular and paracellular pathways [11]. In the present study, we aimed to determine whether ascorbic acid did have other actions in the SVCT1/2-expressing Caco-2 cells by exposing cells to ascorbic acid well before the calcium absorption experiment in Ussing chamber. After being transported into the cells, ascorbic acid was able to exert pro- and/or anti-oxidant actions depending on the intracellular iron level and pH [47]. In the presence of both ascorbic acid and ferric ion, intracellular production of oxygen radicals probably increased through Fenton reaction [48]. However, we found that ascorbic acid did not affect transepithelial calcium transport in FeCl_3_-exposed Caco-2 monolayer. Therefore, the negative effect of ferric ion on 1,25(OH)_2_D_3_ action was rather specific and robust, and was not simply alleviated by generic anti-oxidant like ascorbic acid.

Regarding the limitations, the present study focused on ferric ion rather than ferrous ion (Fe^2+^); therefore, future experiments are required to confirm that both ferrous and ferric ions are able to inhibit the 1,25(OH)_2_D_3_-induced calcium absorption in vivo. Since the iron transporter DMT1 only uptakes ferrous ions, but not ferric ions, a ferric reductase namely Dcytb serves to reduce ferric ions into ferrous ions prior to absorption. In other words, ferrous ions were the majority of ionic iron moving across the apical membrane, and thus ferrous treatment might similarly induce an inhibitory effect on 1,25(OH)_2_D_3_-induced calcium transport. It was noteworthy that exposure to FeCl_3_ for 24–72 h significantly downregulated DMT1 expression in Caco-2 cells, suggesting a compensatory or negative feedback response during excessive iron uptake. Indeed, DMT1 was reportedly modulated by extracellular calcium. Shawki and Mackenzie demonstrated that extracellular calcium was a noncompetitive DMT1 inhibitor, which could reduce cellular iron uptake; however, DMT1 itself did not uptake calcium into the cytoplasm [3].

In conclusion, ferric ion was found to completely diminish the 1,25(OH)_2_D_3_-enhanced calcium transport but not the baseline calcium transport, and could retain its inhibitory action even though cells were no longer in the presence of FeCl_3_ (Fig 5B). The inhibitory action of ferric ion was rapid as demonstrated by its effects on calcium flux and electrical parameters being observed after 20-min exposure (Fig 5A). The finding that ascorbic acid did not increase calcium transport in FeCl_3_-exposed Caco-2 monolayer suggested that it did not have a significant role as a pro- or anti-oxidant under these conditions. Although more investigation is required to reveal the molecular mechanism of ferric ion-induced inhibition of 1,25(OH)_2_D_3_ actions, the present study has provided evidence to help explain how iron diminishes intestinal calcium transport and to support a notion that oral iron and calcium supplement should be given separately to avoid calcium absorption being compromised by iron.

## Supporting information

S1 Fig Effects of ascorbic acid on solubility of CaCO3. Results are expressed as mean ± SE. *P < 0.05; ***P < 0.001 compared with the control group (open circle). ^†^*P* < 0.05 compared with ascorbic acid group (black circle). ^##^*P* < 0.01; ^###^*P* < 0.001 compared with HCl (pH 4) group (black square with dash line).
(EPS)
